# Plant tolerance mechanisms to DNA-damaging UV stress

**DOI:** 10.1093/jxb/eraf272

**Published:** 2025-06-24

**Authors:** Erenay Asik, Syed Zain Kashif, Onur Oztas

**Affiliations:** Department of Molecular Biology and Genetics, College of Sciences, Koc University, Istanbul, 34450, Turkey; Department of Molecular Biology and Genetics, College of Sciences, Koc University, Istanbul, 34450, Turkey; Department of Molecular Biology and Genetics, College of Sciences, Koc University, Istanbul, 34450, Turkey; NC State University, USA

**Keywords:** base excision repair, DNA damage, DNA repair, double strand break repair, flavonoid, nucleotide excision repair, photolyase, translesion synthesis, UV

## Abstract

Due to their sessile lifestyle, plants are continuously exposed to the UV component of sunlight, which threatens their genome stability. Although the Earth's ozone layer prevents a significant portion of the DNA-damaging UV radiation from reaching the surface, it still causes the formation of pyrimidine dimers in the genome that hinder transcription and DNA replication, and also causes the generation of reactive oxygen species (ROS), leading to oxidative DNA damage. To mitigate these effects, plants have evolved an elaborate, multilayered defense system to ensure genome stability under UV stress. Plants contain UV-shielding molecules that function as natural sunscreens to attenuate penetration into deeper tissues, and they also utilize the photoreactivation pathway, in which photolyase enzymes specifically recognize and repair pyrimidine dimers in a manner that is dependent on blue light. They perform light-independent nucleotide excision repairs that excise the pyrimidine dimer-containing oligonucleotides through dual incisions, followed by repair synthesis and ligation. They also maintain DNA replication under UV stress with the aid of translesion synthesis polymerases, which bypass damaged bases. Moreover, to sustain genome stability, DNA damage caused by UV-generated ROS and replication stress is eliminated through base excision repair, which corrects oxidative damage, as well as through pathways for double-strand-break repair, including classical non-homologous end joining, homologous recombination, alternative end joining, and single-strand annealing. Here we provide an overview of the molecular mechanisms that underlie plant UV tolerance. A deeper understanding of these pathways is essential for developing strategies to develop UV-resilient crop varieties.

## Teaser

This review considers the multilayered strategies that plants employ to protect themselves from the detrimental effects of DNA-damaging UV radiation, thereby maintaining the stability of their genomes.

## Introduction

The ozone layer in the stratosphere protects the Earth from the UV component of solar radiation. UV is categorized into three types based on the wavelength, namely UVA (315–400 nm), UVB (280–315 nm), and UVC (100–280 nm). While the Earth's ozone layer effectively absorbs all UVC and most UVB radiation, ∼5% of UVB and all the UVA still reach the surface. The thinning of the ozone layer and the ongoing effects of climate change have caused UVB levels to increase, which negatively influences plant growth and development (Stapleton, 1992; Britt, 1995; Bornman *et al*., 2019; Barnes *et al*., 2023; Chatzopoulou *et al*., 2025). Since plants have a sessile lifestyle and depend on sunlight for photosynthesis, they are continuously exposed to these heightened UVB levels (Bernhard *et al*., 2023; Neale *et al*., 2023).

UVB decreases Rubisco activity and chlorophyll content and impairs PSII, resulting in a reduction in the efficiency of photosynthesis (Vu *et al.*, 1984; Greenberg *et al.*, 1989; Takahashi *et al.*, 2010; Sztatelman *et al.*, 2015). UVB exposure also causes the inactivation and fragmentation of mitochondria (Dündar *et al.*, 2020). Moreover, UVB exposure increases the levels of reactive oxygen species (ROS), which damage RNA, proteins, and lipids (Hideg *et al.*, 2013), and crosslink RNA and ribosomal proteins in maize (Casati and Walbot, 2004). The main effect of UVB on plants is the formation of lesions in the DNA of the nuclear, chloroplast, and mitochondrial genomes. Through ROS production, UV exposure can trigger the accumulation of 8-oxo-7,8-dihydro-2'-deoxyguanosine (8-oxoG) (Watanabe *et al.*, 2006; Balestrazzi *et al.*, 2010), which increases the probability of G→C and T→A substitutions that occur during DNA replication (Cheng *et al.*, 1992). UV radiation can indirectly cause the formation of double-strand breaks (DSBs) in plant genomes, which occur when replication forks stall and collapse at unrepaired UV-induced lesions or from closely spaced single-strand breaks (SSBs) triggered by UV-induced ROS. In addition, nucleotide excision repair of adjacent UV lesions on opposite strands can generate DSB-like intermediates (Ben-Tov *et al.*, 2024).

**Fig. 1.**
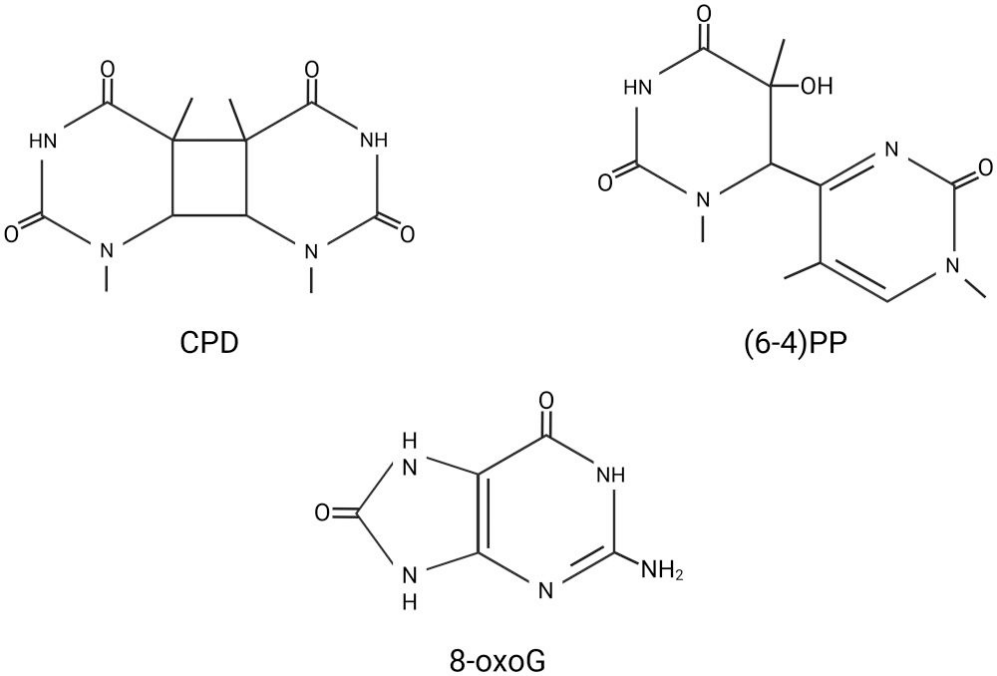
UV-induced DNA lesions. UV radiation directly causes the formation of cyclobutane pyrimidine dimers (CPDs) and pyrimidine (6-4) photoproducts [(6-4)PPs]. CPDs have a cyclobutane ring that connects the C5 and C6 positions of two neighboring pyrimidines, while (6-4)PPs involve a bond between the C6 position of one pyrimidine and the C4 position of the next one. Along with these direct photoproducts, UV exposure also causes the formation of reactive oxygen species that lead to oxidative DNA damage. One of the most common oxidative lesions is 8-oxo-7,8-dihydroguanine (8-oxoG), a mutagenic change to guanine. 8-oxoG keeps the purine ring structure but has a carbonyl group at the C8 position and a hydroxyl group at N7.

UVB exposure causes the formation of dimers between adjacent pyrimidines that block transcription and DNA replication by hindering the elongation of RNA and DNA polymerases (Chan *et al.*, 1985; Rockx *et al.*, 2000; Nakamura *et al.*, 2021). Two major types of UVB-induced DNA lesions exist: pyrimidine–pyrimidone (6-4) photoproducts [(6-4)PPs] and cyclobutane pyrimidine dimers (CPDs). In addition, Dewar photoproducts can also form through photoisomerization of (6-4)PPs (Taylor *et al.*, 1988). While CPDs contain a cyclobutane ring that links the C5 and C6 positions of two neighboring pyrimidines, (6-4)PPs consist of a single covalent bond linking the C6 position of one pyrimidine to the C4 position of the neighboring pyrimidine (Sancar, 2016) (Fig. 1). Due to the structural difference between (6-4)PPs and CPDs, (6-4)PPs cause a more significant distortion and bending of the helix structure of DNA (Kim *et al.*, 1995 ).

UV exposure brings about the formation of more CPDs than (6-4)PPs. The formation of both depends on the dose and wavelength (Dany *et al.*, 2001; Li *et al.*, 2002). The formation of (6-4)PP requires higher energy and therefore it occurs more frequently under UVC exposure compared with UVB. Thus, the CPD:(6-4)PP ratio varies with UV wavelength, and increases under lower-energy UV radiation such as UVB (Perdiz *et al.*, 2000). In addition, the DNA sequence also determines whether CPDs or (6-4)PPs are more likely to form at a genomic location. Both can form at all dipyrimidine sites, but there is variation in their distribution. Both predominantly form at TT dipyrimidine sites; however, (6-4)PPs are also frequently found at TC sites (Hu *et al.*, 2017).

## UVB detection to regulate tolerance mechanisms

**Fig. 2.**
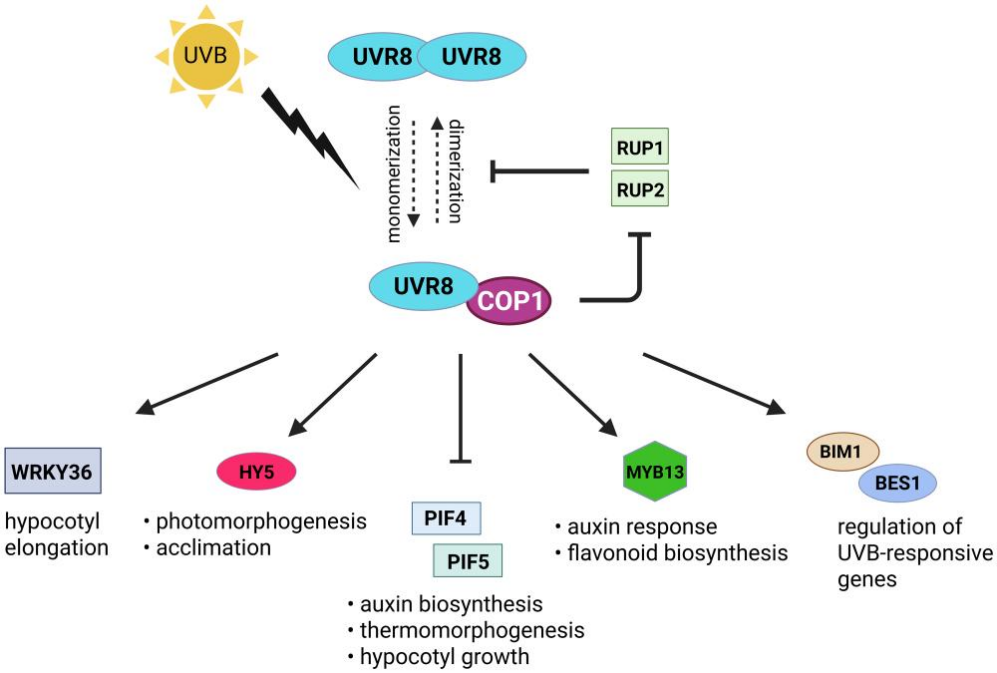
UVB perception and signaling mediated by UV Resistance Locus8 (UVR8). In the absence of UVB, UVR8 remains in the cytosol as an inactive homodimer. When exposed to UVB, it monomerizes and translocates to the nucleus, where it interacts with Constitutively Photomorphogenic1 (COP1). COP1 extends UVR8 monomer activity and sustains UVB signaling by degrading the negative regulators Repressor of UV-B Photomorphogenesis 1 (RUP1) and RUP2, which promote UVR8 redimerization and inactivation. The UVR8–COP1 complex stabilizes Elongated Hypocotyl 5 (HY5), a key regulator of photomorphogenesis and acclimation, and facilitates the degradation of Phytochrome Interacting Factor 4 (PIF4) and PIF5, repressing auxin biosynthesis, thermomorphogenesis, and hypocotyl growth. UVR8 also interacts with MYB13 to regulate the auxin response and flavonoid biosynthesis, with WRKY36 to promote hypocotyl elongation, and with BRI1-EMS-Suppressor 1 (BES1) and BES1-Interacting MYC-Like 1 (BIM1) to modulate UVB-responsive gene expression.

The sensing of UVB radiation through the photoreceptor UV Resistance Locus8 (UVR8) regulates various physiological processes, including auxin signaling, flavonoid biosynthesis, cotyledon expansion, inhibition of hypocotyl growth, stress acclimation, and thermogenesis (Fig. 2). UVR8 is located in the cytosol as a homodimer and upon UVB exposure it dissociates into monomers (Christie *et al.*, 2012; Wu *et al.*, 2012) and translocates to the nucleus where it interacts with Constitutively Photomorphogenic1 (COP1) (Kliebenstein *et al.*, 2002; Kaiserli and Jenkins, 2007; Favory *et al.*, 2009; Rizzini *et al.*, 2011). COP1 degrades Repressor of UV-B Photomorphogenesis 1 (RUP1) and RUP2, which are negative regulators that promote the redimerization and inactivation of UVR8. Their degradation by COP1 prolongs UVR8 monomerization, thereby sustaining UVB signaling (Yin *et al.*, 2015). The UVB-mediated accumulation of the UVR8–COP1 complex in the nucleus stabilizes the transcription factor Elongated Hypocotyl 5 (HY5), which is involved in photomorphogenesis and acclimation to environmental stress (Oravecz *et al.*, 2006; Binkert *et al.*, 2014). Furthermore, the UVR8–COP1 complex leads to the suppression of auxin biosynthesis, thermomorphogenesis, and hypocotyl growth by promoting the degradation of the transcription factors Phytochrome Interacting Factor 4 (PIF4) and PIF5 (Hayes *et al.*, 2014, 2017; Sharma *et al.*, 2019; Tavridou *et al.*, 2020). In addition, UVB-activated UVR8 binds to the MYB13 transcription factor and regulates auxin-response and flavonoid biosynthesis genes (Qian *et al.*, 2020), and interacts with WRKY36 to promote hypocotyl elongation (Yang *et al.*, 2018) and with BRI1-EMS-Suppressor 1 (BES1) and BES1-Interacting MYC-Like 1 (BIM1) that modulate the expression of UVB-responsive genes (Liang *et al.*, 2018).

## The strategies of plants to cope with UV stress on DNA

**Fig. 3.**
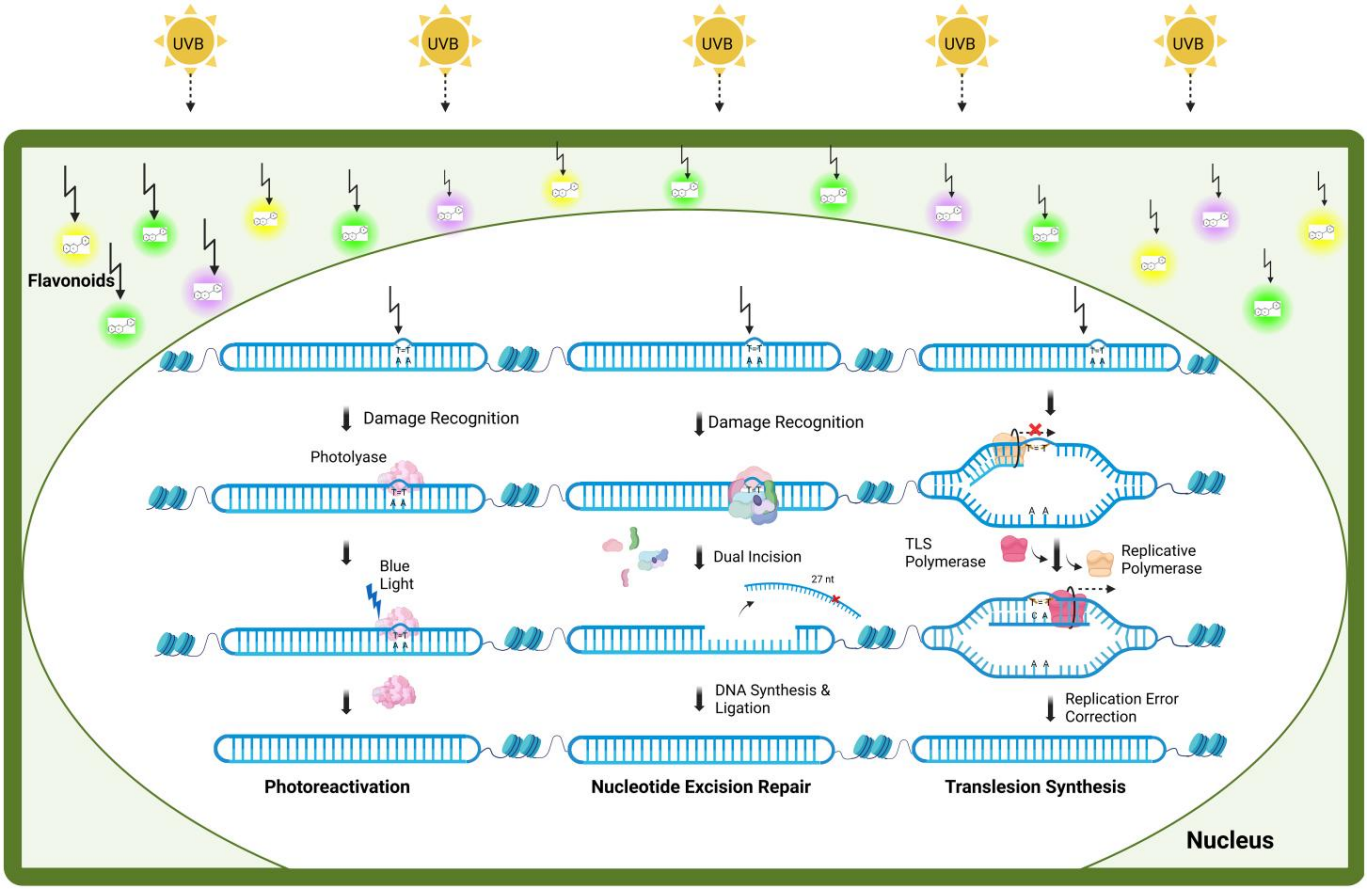
Plant mechanisms to maintain genome stability under UV stress. Plants shield against UVB with the help of flavonoid molecules; however, it still penetrates the nucleus and causes formation of pyrimidine dimers on the genome. These dimers can be eliminated by photoreactivation and nucleotide excision repair. In photoreactivation, photolyase enzymes bind to the pyrimidine dimers and reverse them to the undamaged state using blue light as an energy source. As an alternative mechanism, the nucleotide excision repair pathway can detect the pyrimidine dimers and excise the dimer-containing oligonucleotide (mainly 27 nucleotides in length) by dual incision. The resulting gap is filled by polymerase and ligase activities. To maintain DNA replication in the presence of pyrimidine dimers that block the elongation of replicative DNA polymerase, plants employ translesion synthesis (TLS) polymerases, which can bypass the damage site. However, this process is often error-prone and can result in mutations, and different pathways exist with different levels of mitigation of errors.

Plants employ different strategies to overcome UV-induced DNA damage, which can cause instability by introducing mutations across the genome (Willing *et al.*, 2016). They protect their DNA through the accumulation of flavonoids, repair DNA damage by photoreactivation and nucleotide excision repair, and employ translesion synthesis polymerases to enable DNA replication to continue despite the presence of pyrimidine dimers (Fig. 3). In addition to causing direct DNA damage, UVB radiation also induces indirect effects on the plant genome, including oxidative lesions and DSBs. These forms of damage are repaired through the base excision repair pathway and DSB repair mechanisms.

### UV-shielding molecules to block UV penetration

Plants protect themselves from the effects of UV radiation by synthesizing a variety of flavonoid phenolics that absorb the UV wavelengths, which mainly accumulate in the epidermal and mesophyll cells of leaves, in the petals of flowers, and in trichomes (Li *et al.*, 1993; Stapleton *et al.*, 1997; Bieza and Lois, 2001; Ferreyra *et al.*, 2021). Species, tissue type, the developmental stage of the plant, and UV exposure time and dose all influence the type and amount of flavonoids that are produced (Agati *et al.*, 2011; Neugart *et al.*, 2013; Ghasemzadeh *et al.*, 2016; Ma *et al.*, 2018). They are synthesized in the cytoplasm through the phenylpropanoid pathway by using phenylalanine as the precursor molecule and then transported to the nucleus, vacuole, and cell wall (Mueller *et al.*, 2000; Feucht *et al.*, 2004; Saslowsky *et al.*, 2005; Marinova *et al.*, 2007). UVB exposure induces the expression of the genes encoding the enzymes of the phenylpropanoid pathway, thereby increasing the levels of UV-shielding flavonoids under high-UVB conditions (Pandey *et al.*, 2019).

**Fig. 4.**
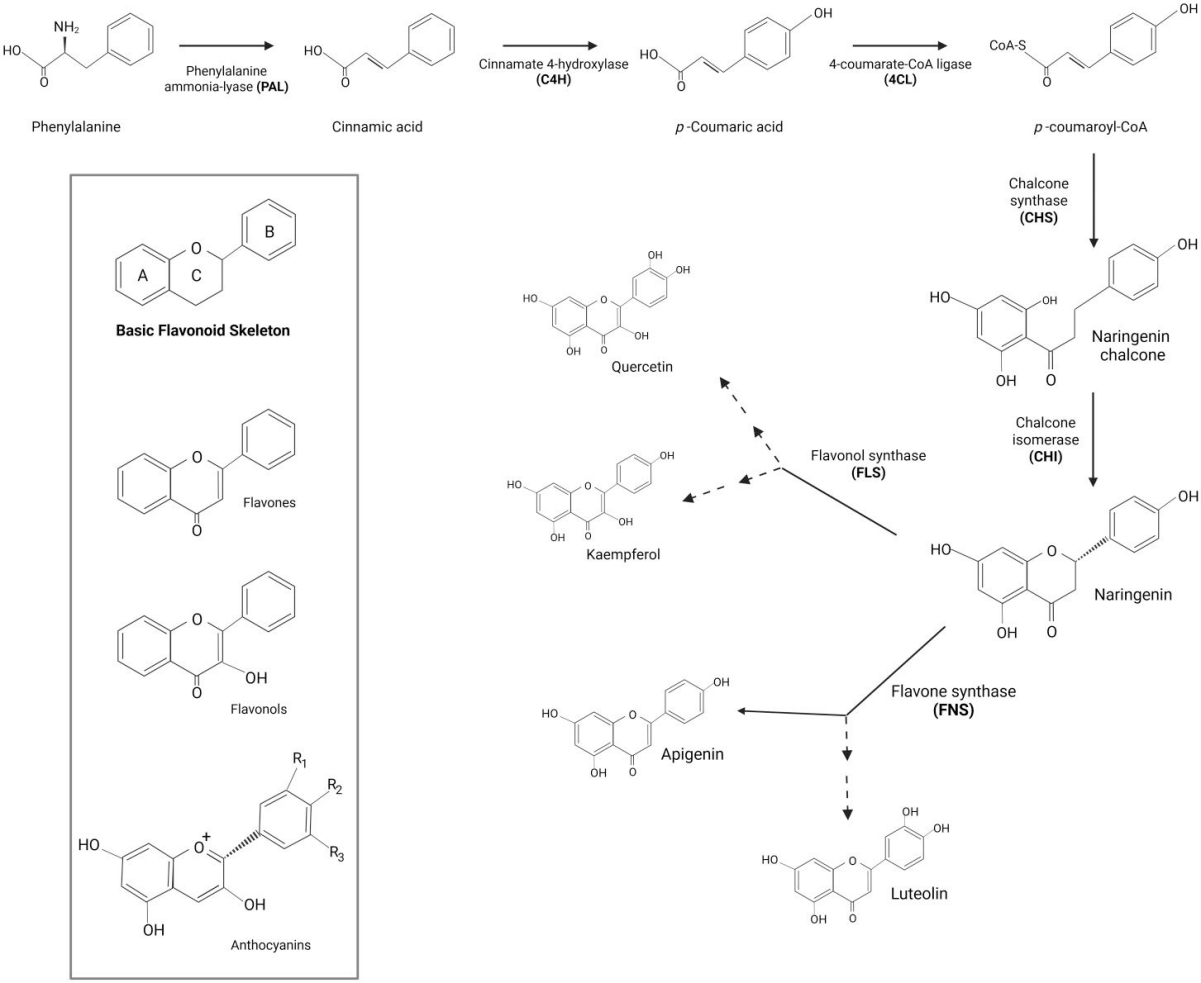
Flavonoid biosynthesis pathway. Phenylalanine is converted to cinnamic acid by PAL, then to *p*-coumaric acid by C4H, and finally to *p*-coumaroyl-CoA by 4CL. The CHS and CHI enzymes catalyse the formation of naringenin, a key precursor for various flavonoids. All flavonoids have a common C_6_-C_3_-C_6_ skeleton with two aromatic rings (A and B) and a heterocyclic C ring.

The biosynthesis of various flavonoid types shares a common pathway (Fig. 4). First, phenylalanine is converted into cinnamic acid by Phenylalanine Ammonia-Lyase (PAL). Cinnamic acid is further hydroxylated to *p*-coumaric acid by Cinnamate 4-Hydroxylase (C4H) and then converted to *p*-coumaroyl-CoA by 4-Coumarate-CoA Ligase (4CL). *p*-Coumaroyl-CoA is the substrate for Chalcone Synthase (CHS), which catalyses its condensation with three molecules of malonyl-CoA to produce naringenin chalcone. This intermediate is then converted into naringenin by Chalcone Isomerase (CHI). Different branches of the pathway are then followed to produce diverse flavonoid compounds from naringenin. These flavonoids possess a C_6_-C_3_-C_6_ carbon backbone (Hahlbrock and Scheel, 1989), including two aromatic rings (A and B) linked with a three-carbon bridge, forming a heterocyclic oxygen-containing ring (C ring). The A ring is derived from the benzo-γ-pyrone structure, while the B ring represents the phenyl ring. The central C ring, which includes the oxygen atom, forms the γ-pyrone ring, that plays a crucial role in determining the chemical properties and classification of flavonoids. The main UV-absorbing flavonoids are flavones, flavonols, and anthocyanins, all of which include the attachment of the B ring to the C2 of the C ring in their structures.

The levels of two flavonols, quercetin and kaempferol, increase in leaves upon UV exposure, and contribute to enhanced tolerance. Quercetin shows a higher accumulation in response to UV radiation than kaempferol, suggesting its more significant role in protection (Ryan *et al.*, 1998; Agati *et al.*, 2011). In maize, UVB increases the expression of two Flavonol Synthase (FLS) enzymes, which share a high degree of sequence identity. One of them, FLS1, has been shown to reduce UV-induced CPD damage (Falcone Ferreyra *et al.*, 2012; Emiliani *et al.*, 2013). The flavonol compositions of two *Vicia faba* accessions from habitats with different UVB levels show variations (Yan *et al.*, 2019). An accession from the Andean region of Colombia and Ecuador that is adapted to growing at high UVB produces higher levels of quercetin glycosides than an accession from Sweden. Quercetin glycosides are characterized by the attachment of glucose molecules to the flavonol backbone, and the glycosylation patterns, which determine UVB-absorbing capacity, also differ between the two accessions. Another class of flavonols, saiginols, are synthesized by Flavonol-Phenylacyltransferase 2 (FPT2) and accumulate in floral tissues to increase UV light tolerance (Tohge *et al.*, 2016). In addition, flavones, mainly apigenin and luteolin, also protect plants from UV exposure. Flavone Synthase (FNS) enzymes catalyse the conversion of naringenin to apigenin. UVB-inducible FNSI and FNSII enzymes in maize, FNS1 in moss, and FNSI in mulberry all increase tolerance by reducing the formation of UVB-induced DNA damage (Righini *et al.*, 2019; H. Li *et al*., 2020; Wang *et al.*, 2020 ). In barley, UV-mediated induction of flavone O-glycosides results in reduced DNA damage (Schmitz-Hoerner and Weissenböck, 2003). In rice, FLAVONE 7-O-GLUCOSYLTRANSFERASE and FLAVONE 5-O-GLUCOSYLTRANSFERASE mediate the glycosylation of flavones and increase the production of various flavone-O-glucosides, thereby playing a role in enhancing UV tolerance (Peng *et al.*, 2017). In addition, maize accumulates two flavone C-glycosides in the leaves and floral tissues under UVB exposure, namely maysin and rhamnosylisoorientin (Casati and Walbot, 2005). Anthocyanins reduce UV penetration into deeper tissues, and UVB regulates their synthesis through the UVR8-mediated pathway (Dębski *et al.*, 2016; H. Li *et al*., 2018; W. Li et al., 2020). In apple, UVB exposure induces the expression of the MdWRKY72 transcription factor, which induces anthocyanin biosynthesis genes (Hu *et al.*, 2020). In maize, the presence of anthocyanins reduces the accumulation of CPDs and (6-4)PPs, protecting plants from UV-induced DNA damage (Stapleton and Walbot, 1994). In addition, hydroxycinnamates, a group of non-flavonoid phenolics, absorb UV wavelengths and prevent the detrimental effects of UV in Arabidopsis seedlings (Kusano *et al.*, 2011).

### Photoreactivation

Despite the presence of UV-absorbing compounds in plant cells, UVB can still induce the formation of pyrimidine dimers in the genome. To cope with these detrimental effects, plants employ a second strategy to sustain growth, namely the repair of UV-induced DNA damage (Jiang *et al.*, 1997; Landry *et al.*, 1997; Hidema *et al.*, 2007). One such repair mechanism is photoreactivation, which relies on photolyase enzymes activated by blue light and is predominantly active in non-proliferating cells (Kimura *et al.*, 2004; Sancar, 2016). Different photolyase enzymes repair CPD and (6-4)PP damage: a single photolyase cannot repair both types of lesions (Sancar, 2003). For example, in Arabidopsis, ULTRAVIOLET HYPERSENSITIVE 2 (UVR2; (also known as PHR1) only repairs CPDs, while UVR3 is responsible solely for the repair of (6-4)PPs (Ahmad *et al.*, 1997; Jiang *et al.*, 1997; Nakajima *et al.*, 1998). Several CPD and (6-4)PP photolyases have been discovered in various species. The first plant photolyase genes were identified in Arabidopsis and white mustard, and demonstrated a high degree of conservation with photolyases from other organisms (Batschauer, 1993; Ahmad *et al.*, 1997; Jiang *et al.*, 1997). Subsequent studies broadened the repertoire of photolyases in diverse species, including rice, and cucumber, emphasizing the widespread presence of both CPD and (6-4)PP photolyases across monocots and dicots (Hada *et al.*, 2000; Takahashi *et al.*, 2002; Hirouchi *et al.*, 2003; Hidema *et al.*, 2007).

The photoreversal of pyrimidine dimers by DNA photolyase begins with base-flipping, in which the UV photoproduct is displaced from the DNA helical axis and extruded into the active site of the enzyme, followed by a light-independent interaction between the substrate-binding cavity of photolyase and the pyrimidine dimers (Vande Berg and Sancar, 1998; Knips and Zacharias, 2017). The photoantenna (also referred to as the second chromophore), which can be 5,10-methenyltetrahydrofolate (MTHF) or 8-hydroxydeazaflavin (8-HDF), absorbs blue-light photons (350–450 nm) and transfers the excitation energy to the catalytic chromophore, which is reduced FAD (FADH^−^). This leads to the transfer of an electron from FADH^−^ to the dimer, converting the pyrimidine dimer to the undamaged state. The electron is then transferred back to regenerate FADH^−^ (Sancar, 1994). The CPD photolyase enzyme in Arabidopsis, UVR2, contains FADH⁻ as the catalytic chromophore and a reduced pterin, most likely MTHF, as the second chromophore (Kleiner *et al.*, 1999; Waterworth *et al.*, 2002). Rice and wheat CPD photolyases are phosphorylated, and Arabidopsis UVR3 includes a phosphorylation motif, implying potential regulation of these enzymes by phosphorylation (Teranishi *et al.*, 2008; Hitomi *et al.*, 2009). UVB, UVA, and blue light regulate the expression of UVR2; however, UVR3 expression is constitutive and does not depend on UVB (Waterworth *et al.*, 2002; N. Li *et al*., 2015).

In addition to repairing pyrimidine dimers in the nucleus, photolyases are also involved in the removal of UV-induced damage in mitochondria and chloroplasts (Draper and Hays, 2000). Exposure to white light has been shown to enhance the repair of CPD lesions in the mitochondria and chloroplasts of maize (Stapleton *et al.*, 1997). In rice, CPD photolyase contains a signal sequence that targets the enzyme to both mitochondria and chloroplasts, where it actively repairs CPD damage (Takahashi *et al.*, 2011, 2014). In addition, UVR3 is localized and functional in mitochondria and chloroplasts (Katarzyna Banas *et al.*, 2018; Zgłobicki *et al.*, 2024).

### Nucleotide excision repair

**Fig. 5.**
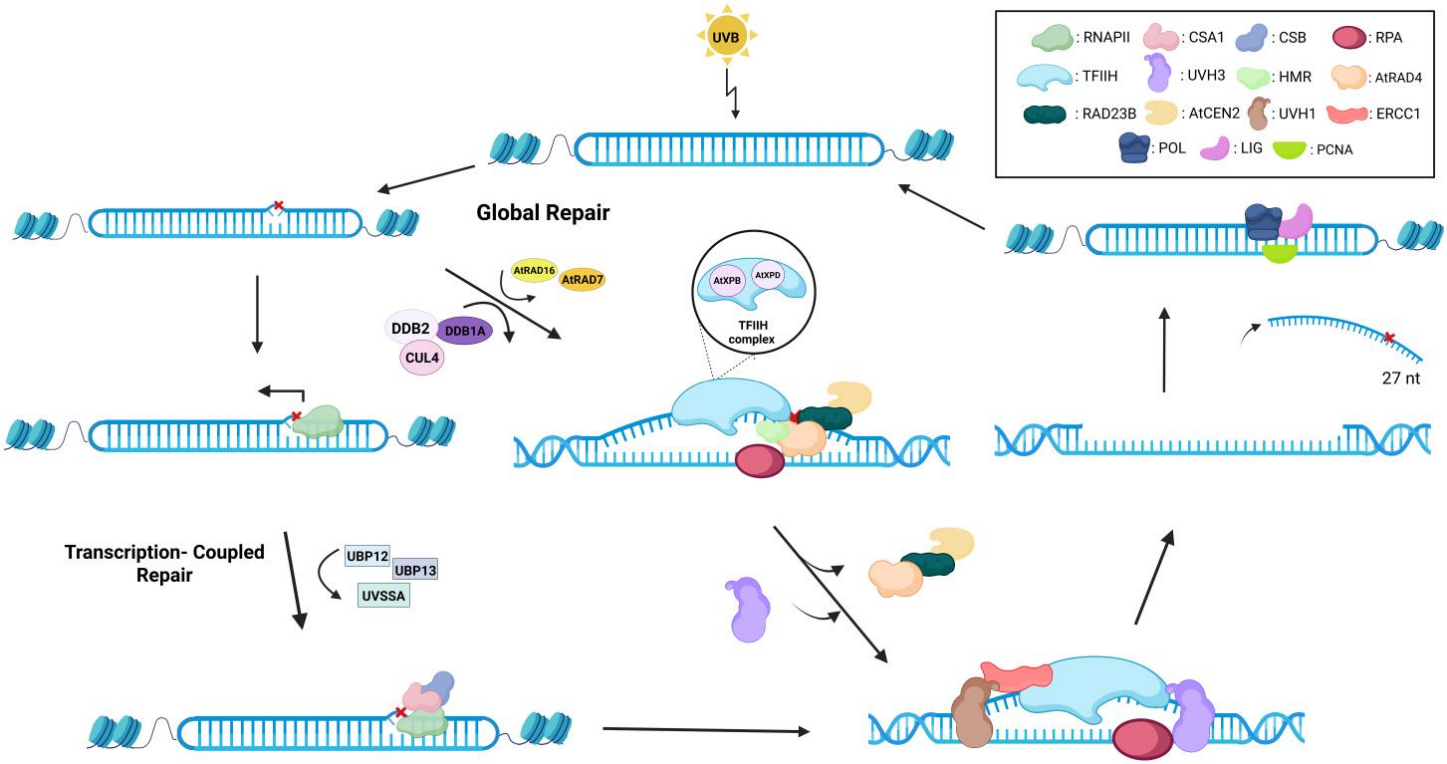
Nucleotide excision repair in plants. In global repair, which occurs throughout the genome, the pyrimidine dimers are detected by the cooperative action of the AtRAD4-RAD23B-AtCEN2 complex, RPA, UVH3 endonuclease, and kinetic proofreading by the TFIIH complex. AtRAD4 interacts with the HMR protein. The TFIIH complex, composed of AtXPB proteins and AtXPD, is then recruited to the damage site, where it unwinds the DNA around the lesion, allowing RPA to bind the undamaged strand. Then, the UVH1 and UVH3 endonucleases excise a pyrimidine dimer-containing oligonucleotide (mainly 27 nucleotides in length) through dual incisions occurring 4–6 nucleotides and 18–21 nucleotides from the 3′- and 5′-end of the DNA adduct, respectively. The actions of polymerases and ligases further synthesize the new strand. The binding of the AtRAD4 complex to the damage site is regulated by the UV-DDB complex, which consists of the DDB1A, DDB2, and CUL4 proteins. The AtRAD16 and AtRAD7 proteins are also involved in global repair. Transcription-coupled repair occurs only when RNAPII stalls at the damage site, which is detected by CSB proteins. CSA is then recruited to the damage site and binds to the CSB protein. The UVSSA, UBP12, and UBP13 proteins also regulate CSB binding to the stalled RNAPII at the damage site. The following steps in transcription-coupled repair are similar to those of global repair. See text for abbreviations.

UV-induced pyrimidine dimers are bulky DNA adducts that distort the double-helix structure. The nucleotide excision repair pathway functions independently of light, unlike photoreactivation, and can efficiently remove bulky DNA adducts, including CPD and (6-4)PP lesions, from the genome at any time in plants (Howland, 1975; Quaite *et al.*, 1994; Canturk *et al.*, 2016; Schalk *et al.*, 2017). Nucleotide excision repair is divided into two sub-pathways, namely global repair and transcription-coupled repair (TCR) (Fig. 5). Global repair removes UV-induced DNA damage from all genomic regions, while TCR occurs explicitly at the transcribed strands of active genes (Fidantsef and Britt, 2011; Oztas *et al.*, 2018).

The development of the eXcision Repair-sequencing (XR-seq) method has enabled the generation of genome-wide maps of nucleotide excision repair, enabling the analysis of genome-wide profiles and dynamics across different organisms for various bulky DNA adducts (Hu *et al.*, 2015, 2018; Adebali et al., 2017; Li *et al.*, 2017; Deger *et al.*, 2022; Kose *et al.*, 2024b; Wu *et al.*, 2024). XR-seq analysis in Arabidopsis has revealed that primary excision products range from 23–27 nucleotides (nt) in length. These products are generated by dual incisions occurring 4–6 nt and 18–21 nt from the 3′- and 5′-end of the pyrimidine dimers, respectively, as indicated by the nucleotide frequency profile across the 27 nt excision products. In addition, a group of shorter fragments ranging from 10–22 nt is detected, that might result from degradation of the excision products from the 5′-end (Huang *et al.*, 1992; Oztas *et al.*, 2018; Kaya *et al.*, 2024). Based on a genome-wide analysis of nucleotide excision repair in Arabidopsis, CPD repair levels are approximately five times higher in the transcribed strands of genes compared to the non-transcribed strands (Oztas *et al.*, 2018), indicating that TCR plays a significant role in CPD removal mediated by nucleotide excision repair, with the transcription rate being the primary determinant of the repair rate. The TCR level in a gene during CPD repair is directly proportional to its transcription rate. Since the circadian clock controls transcription of most genes in Arabidopsis, TCR accordingly exhibits a circadian oscillation pattern in the plant. The strong dependence of nucleotide excision repair on the transcriptional state indicates that plants use it to target DNA repair machinery to the genes via TCR to maintain transcription under UVB stress. The bias in repair within specific genomic regions has not yet been observed for photoreactivation. In contrast, TCR contributes minimally to the repair of (6-4)PP lesions that is mediated by nucleotide excision repair, with its activity being nearly absent, suggesting that TCR-mediated bias is not necessary for (6-4)PP repair, most likely due to the faster repair rate of (6-4)PP lesions compared to CPD lesions (Oztas *et al.*, 2018). However, the repair of (6-4)PP lesions peaks at both the transcription start and end sites of genes, indicating preferential repair at these regulatory genomic regions. Moreover, the chromatin state influences the repair of both CPD and (6-4)PP lesions that is mediated by nucleotide excision repair, as it is less efficient in heterochromatic regions than in open chromatin regions (Oztas *et al.*, 2018; Kaya *et al.*, 2024). Thus, UV-induced lesions are more enriched in heterochromatin regions, and their repair requires chromatin remodeling to allow access of repair proteins to the damage site and to restore the chromatin structure after DNA repair, a process that is dependent on the UVR8 photoreceptor (Johann to Berens *et al.*, 2023).

Plants possess many homologs of the nucleotide excision repair components found in mammals. In mammalian nucleotide excision repair, UV-induced DNA lesions are detected by co-operative interaction between Xeroderma Pigmentosum Complementation Group C (XPC) complex, including the Human Rad23 Homolog B (HR23B) and Centrin-2 (CEN2) proteins, Xeroderma Pigmentosum Group A (XPA), Replication Protein A (RPA), XPG, and kinetic proofreading by the Transcription Factor IIH (TFIIH) complex (Reardon and Sancar, 2003; Lindsey-Boltz *et al.*, 2023). Upon binding to the lesion, the XPC complex recruits the TFIIH complex and XPA to the lesion site to verify the damage. TFIIH and XPA form a lesion-containing DNA bubble, with RPA binding to the undamaged strand. The TFIIH complex, containing XPB and XPD helicases, unwinds the DNA toward the lesion. This is followed by dual incisions around the damage, carried out by the endonucleases XPF–Excision Repair Cross-Complementation group 1 (ERCC1) and XPG, resulting in the removal of the lesion-containing oligonucleotide. The resulting DNA gap is then filled by DNA polymerases and sealed by DNA ligases, replacing the damaged oligonucleotide with a new, undamaged DNA fragment. This mechanism, known as global repair, is a sub-pathway of excision repair that removes UV-induced damage from all genomic regions (Mu *et al.*, 1995, 1996; Sijbers *et al.*, 1996; Evans *et al.*, 1997; Nocentini *et al.*, 1997; Sugasawa *et al.*, 1998; Araújo *et al.*, 2000; Yokoi *et al.*, 2000; Araki *et al.*, 2001; Rademakers *et al.*, 2003; Coin *et al.*, 2007; Ogi *et al.*, 2010; Sancar, 2016).

The recognition of UV-induced DNA lesions within the chromatin environment requires the UV-Damaged DNA-Binding (UV-DDB) complex, a heterodimer of DDB1 and DDB2 proteins, and the XPC complex. DDB1 and DDB2 associate with Cullin 4 (CUL4) to form an E3 ubiquitin ligase complex to facilitate the recruitment of XPA to the damage site and promote its association with damaged DNA (Nichols *et al.*, 2000; Wakasugi *et al.*, 2002; Wang *et al.*, 2004). When UV lesions occur on the transcribed strands of active genes, elongating RNA Polymerase II (RNAPII) stalls at the damage site, and this is detected by the Cockayne Syndrome group B (CSB) protein, leading to the recruitment of CSA and CSB ubiquitination. In addition, the UV-Stimulated Scaffold Protein A (UVSSA) interacts with Ubiquitin-Specific Protease 7 (USP7) to form a complex that deubiquitinates and stabilizes the CSB-RNAPII complex. To allow nucleotide excision repair factors to access and remove the transcription-blocking lesion, the stalled RNAPII is displaced from the DNA template to rescue the transcription (van Gool *et al.*, 1997; Nakazawa *et al.*, 2012; Schwertman *et al.*, 2012; Zhang *et al.*, 2012; Chiou *et al.*, 2018; Selby *et al.*, 2023). Yeast nucleotide excision repair involves the Radiation Sensitive 7 (RAD7)–RAD16 complex, which is absent in mammalian nucleotide excision repair. This complex plays a key role in repairing UV-induced DNA lesions in non-transcribed DNA strands and transcriptionally inactive regions (Verhage *et al.*, 1994; Wang *et al.*, 1997; Reed *et al.*, 1998).

The molecular mechanism of nucleotide excision repair in plants is less well understood than that of bacteria, yeast, and mammals. Plants possess homologs of repair factors from mammals and yeast, with some of these genes duplicated in plant genomes. Plants lack the XPA protein, which is essential for efficient nucleotide excision repair in mammals (Singh *et al.*, 2010). Even though nucleotide excision repair can occur in humans, worms, and flies in the absence of XPA, its efficiency is significantly reduced in these organisms (Kose *et al.*, 2024*a*). The duplication of genes encoding DDB1, CSA, XPB, and HR23B and the highly efficient nucleotide excision repair activity despite the absence of XPA indicate that plants have evolved a unique pathway that compensates for the absence of certain canonical factors in mammalian cells. However, the specific molecular adaptations and functional roles of the duplicated repair factor genes in plants remain largely unclear, emphasizing a significant gap in our understanding of how nucleotide excision repair is orchestrated in the plant kingdom.

The components of the XPC complex are conserved in plants. In Arabidopsis, RADIATION-SENSITIVE 4 (RAD4), the homolog of human XPC, interacts with CEN2 and RAD23B, one of the four RAD23 proteins. Arabidopsis plants lacking RAD4, CEN2, or RAD23 proteins become hypersensitive to UVC. Arabidopsis RAD4 also interacts with the RAD23-like protein Hemera (HMR), the expression of which partially rescues the UV sensitivity of yeast *rad23* mutants. In Arabidopsis, the absence of HMR causes increased UV sensitivity (Molinier *et al.*, 2004; Liang *et al.*, 2006; Chen *et al.*, 2010; Farmer *et al.*, 2010; Lahari *et al.*, 2017). Two RAD23 isoforms have been identified in carrot (*Daucus carota*), that are capable of complementing a UV-sensitive *rad23* mutant in yeast (Sturm and Lienhard, 1998).

Arabidopsis has two homologs of DDB1, namely DDB1A and DDB1B. DDB2 and DDB1A interact with CUL4, forming a CUL4-DDB1A-DDB2 protein complex that plays a crucial role in the global repair of UV-induced DNA lesions. Arabidopsis plants lacking CUL4, DDB1A, or DDB2 exhibit hypersensitivity to UVC radiation, while overexpression of *DDB1A* enhances UV resistance. This increased resistance is consistent with the improved removal of CPDs and (6-4)PPs in *DDB1A*-overexpressing plants, whereas *ddb1a* mutant plants show reduced repair efficiency compared to the wild type. Moreover, DDB2 is localized in the nucleus, whereas DDB1A is primarily found in the cytosol but accumulates in the nucleus upon UVC exposure. The expression of both *DDB1A* and *DDB1B* increases in response to UV radiation (Koga *et al.*, 2006; Molinier *et al.*, 2008; Al Khateeb and Schroeder, 2009). Furthermore, Arabidopsis has two XPB homologs, XPB1 and XPB2, that share 95% amino acid identity and contribute to increased UV tolerance. While the expression of both genes is regulated by light, only XPB1 transcription is specifically up-regulated in response to UVB radiation (Liu *et al.*, 2000; Costa *et al.*, 2001; Morgante *et al.*, 2005; Spampinato, 2017; Masuda *et al.*, 2020). Furthermore, Arabidopsis XPD interacts with a component of the TFIIH complex, p44, both of which are homologs of the respective proteins in humans. XPD in Arabidopsis plays a key role in removing (6-4)PP lesions by nucleotide excision repair, and a lack of *XPD* expression leads to increased sensitivity to UV radiation (Seroz *et al.*, 2000; Liu *et al.*, 2003). Moreover, the Arabidopsis UVH1/RAD1 protein, a homolog of the human XPF, and UVH3/UVR1, a homolog of the human XPG, are essential for UV tolerance and are required for efficient nucleotide excision repair (Harlow *et al.*, 1994; Fidantsef *et al.*, 2000; Gallego *et al.*, 2000; Liu *et al.*, 2000, 2001, 2003). Furthermore, Arabidopsis has two homologs of yeast RAD16, namely RAD16 and RAD16b, which contribute to UV tolerance and growth, and the *rad16* and *rad16b* null mutants exhibit increased UV sensitivity. Moreover, overexpression of *RAD16* increases UV tolerance. The physical interaction between RAD16 and RAD7 suggests that the Arabidopsis RAD7-RAD16 complex is involved in plant nucleotide excision repair (Weber *et al.*, 2005; Shaked *et al.*, 2006; Lahari *et al.*, 2018; Alrayes *et al.*, 2023). Moreover, Arabidopsis possesses two homologs of CSA, namely CSA1 and CSA2. While CSA1 is essential for TCR, CSA2 has a minor influence (Kaya *et al.*, 2022). These proteins have been shown to form heterotetramers, localize to the nucleus, and interact with DDB1A. The lack of both CSA1 and CSA2 causes increased sensitivity to UVB radiation (Biedermann and Hellmann, 2010; Zhang *et al.*, 2010). In addition, Arabidopsis possesses two homologs of CSB, CHR8 and CHR24, the absence of which causes UV sensitivity (Shaked *et al.*, 2006). Arabidopsis plants lacking the UVSSA and USP7 homologs UBP12 and UBP13 also exhibit increased UV sensitivity (Al Khateeb *et al.*, 2019).

### Translesion synthesis to sustain DNA replication under UV stress

Even though UV-shielding molecules and DNA repair mechanisms remove UV-induced DNA damage, they do not immediately eliminate all lesions. Translesion synthesis (TLS) polymerases can bypass UV-induced lesions to maintain DNA replication under UV stress. However, if not corrected by the exonuclease activity of DNA polymerases or mismatch repair, the errors caused by low-fidelity TLS polymerases can lead to mutations in the genome, including base substitutions and frameshifts. During DNA synthesis, a replicative DNA polymerase stalls at the damage site in the presence of pyrimidine dimers. As a response, Y-family polymerases, including TLS polymerases, interact with Proliferating Cell Nuclear Antigen (PCNA) to catalyse TLS. To bypass the damage, a TLS polymerase temporarily takes over from replicative DNA polymerase and inserts one or more nucleotides opposite the damage. While this process often results in the incorporation of incorrect nucleotides, it sustains replication fork progression.

In Arabidopsis, two distinct TLS pathways respond to UV-induced DNA damage: a more error-prone pathway involving Polζ and Rev1 and a less mutagenic pathway mediated by Polη. Polζ and Rev1 facilitate the error-prone bypass of both CPD and (6-4)PP, while Polη, interacting with PCNA1 and PCNA2, can bypass CPD lesions but not (6-4)PP. The *REV3* gene encodes the catalytic subunit of DNA polymerase ζ (Polζ), and DNA replication in the root meristem of the Arabidopsis *rev3* mutant is significantly decreased after UVB irradiation. *REV7* encodes a regulatory subunit of Polζ, while *REV1* encodes a Y-family polymerase. Under chronic UVB exposure, *rev1* and *rev7* mutants exhibit reduced growth compared to wild type plants, highlighting the importance of these genes in UV tolerance (Sakamoto *et al.*, 2003; Takahashi *et al.*, 2005; Santiago *et al.*, 2006; Curtis and Hays, 2007; Anderson *et al.*, 2008; Hoffman *et al.*, 2008; Nakagawa *et al.*, 2011).

### Base excision repair

Exposure to UV light triggers the production of ROS in cells, leading to oxidative DNA damage, which is marked by chemical changes to DNA bases such as the formation of formamidopyrimidines (Fapy), thymine glycol (Tg), and 8-oxoG (Fig. 1), and the further breakdown products of the latter guanidinohydantoin (Gh) and spiroiminodihydantoin (Sp) (Watanabe *et al.*, 2006; Greene *et al.*, 2007). 8-oxoG can pair with either C or A during replication, and can cause G-to-T transversions when paired with A (Shibutani *et al.*, 1991; Moriya, 1993). Fapy•dG exhibits slightly greater mutagenic potential than 8-oxoG. Fapy lesions cause similar challenges for DNA polymerases and repair enzymes as those posed by 8-oxoG (Greenberg, 2012). Tg is an oxidative lesion of thymine that can give rise to T-to-C transition mutations (Essigmann *et al.*, 1989). These oxidative DNA lesions are mainly repaired through base excision repair (BER) in plants. BER functions through two pathways based on the repair patch size: short-patch BER, which substitutes only the damaged nucleotide, and long-patch BER, which entails synthesizing a DNA segment of two or more nucleotides (Córdoba-Cañero *et al*., 2009). The damaged bases from DNA are detected by lesion-specific enzymes called DNA glycosylases, which can be either monofunctional or bifunctional. When a monofunctional DNA glycosylase initiates BER, it hydrolyses the N-glycosidic bond between the damaged base and the sugar-phosphate backbone, generating an apurinic/apyrimidinic (AP) site containing an intact 2′-deoxyribose lacking its nitrogenous base. This site is then processed by Apurinic/Apyrimidinic Endonuclease 1 (APE1), which cleaves the DNA backbone at the 5′-side of the AP site. The result is a single-nucleotide gap flanked by a 3′-hydroxyl group and a 5′-deoxyribose phosphate (dRP) residue. When bifunctional DNA glycosylases are involved, these enzymes not only excise the damaged base but also possess AP lyase activity that cleaves the DNA backbone 3′ to the AP site via a β-elimination reaction. This cleavage generates a SSB characterized by a 3′-α,β-unsaturated aldehyde and a 5′-phosphate terminus. A subset of bifunctional glycosylases, known as β,γ-lyases, further catalyses a γ-elimination reaction that removes the entire sugar moiety, resulting in a single-nucleotide gap flanked by phosphate groups at both the 3′- and 5′-ends. The accessory factors Poly(ADP-Ribose) Polymerase 1 (PARP-1) and X-Ray Repair Cross-Complementing 1 (XRCC1) bind at the gap and promote repair (Roldán-Arjona *et al.*, 2019; Grin *et al.*, 2023). In short-patch BER, the 5′-dRP group is excised by DNA Polymerase β or λ (POLβ or POLλ), which subsequently engage in gap-filling DNA synthesis. This is followed by ligation mediated by DNA ligase I or III. In contrast, long-patch BER involves strand-displacement DNA synthesis led by POLβ or other polymerases. The 5′-flap created in this process is removed by Flap Endonuclease 1 (FEN1), generating a nicked DNA intermediate for ligation (Matsumoto and Kim, 1995; Frosina *et al.*, 1996; Kubota *et al.*, 1996; Klungland and Lindahl, 1997; Prasad *et al.*, 2000; Lindahl, 2016; Beard *et al.*, 2019). Furthermore, one strategy to prevent 8-oxoG from integrating into the genome is by using 8-oxodGTPase enzymes, such as MutT in *E. coli* and MutT Homolog 1 (MTH1) in humans. These enzymes sanitize the nucleotide pool by hydrolysing 8-oxo-dGTP, which inhibits its misincorporation by DNA polymerases during replication. In Arabidopsis, the Nudix Hydrolase NUDX1 serves a similar role by hydrolysing 8-oxo-dGTP, thereby sustaining genome integrity through the sanitization of the nucleotide pool (Yoshimura *et al.*, 2007).

Plants possess homologs of the bacterial Formamidopyrimidine-DNA Glycosylase (Fpg) and the eukaryotic 8-Oxoguanine glycosylase (OGG1) enzyme, which are involved in BER-mediated removal of oxidative base lesions (Scortecci *et al.*, 2007; Macovei *et al.*, 2011). In Arabidopsis, the Fpg homolog MMH-1 and OGG1 are responsible for removing 8-oxoG, Gh, Sp, and Faby oxidative lesions, thereby inhibiting G-to-T transversions. Both are bifunctional enzymes that exhibit DNA glycosylase and AP lyase activities. The subsequent processing of the 3'-blocking termini produced by these enzymes requires the concerted action of the zinc finger DNA 3'-Phosphoesterase (ZDP) and the Apurinic/Apyrimidinic Endonuclease Redox Protein (ARP) (Ohtsubo *et al.*, 1998; Gao and Murphy, 2001; Morales-Ruiz *et al.*, 2003; Murphy and George, 2005; Kathe *et al.*, 2009; Murphy and Guo, 2010; Córdoba-Cañero *et al.*, 2014). Arabidopsis NTH1 and NTH2 also act as bifunctional DNA glycosylases, and are capable of efficiently recognizing and excising thymine glycol from DNA (Roldán-Arjona *et al.*, 2000). Following the excision of oxidative DNA lesions by DNA glycosylases, an AP site is generated. An AP endonuclease then cleaves the DNA phosphodiester backbone at this site, producing a strand break that is suitable for repair by DNA polymerase and ligase. The ARP protein in Arabidopsis acts as a class II AP endonuclease, and this represents the major AP endonuclease activity in plant cell extracts. In addition, APE2 and APE1L exhibit AP endonuclease activities (Babiychuk *et al.*, 1994; Córdoba-Cañero *et al.*, 2011; Y. Li *et al*., 2015; J. Li *et al*., 2018).

**Fig. 6.**
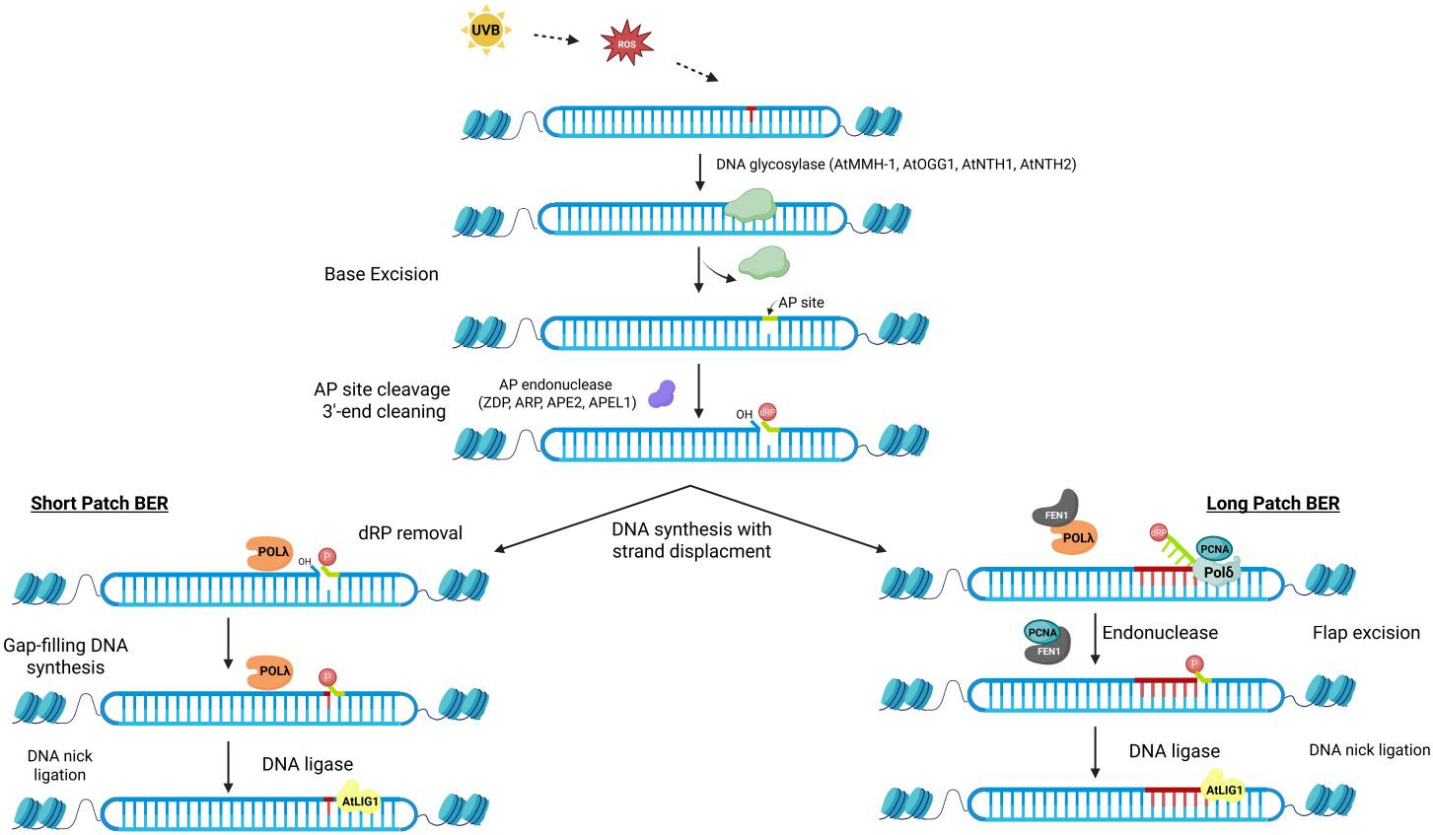
Base excision repair (BER) in plants. AtMMH-1 and AtOGG1 are bifunctional DNA glycosylases with apurinic/apyrimidinic (AP) lyase activity, excising oxidative DNA lesions and producing 3′-blocking termini that ZDP and ARP process. AtNTH1 and AtNTH2 also function as bifunctional glycosylases to eliminate thymine glycol. ARP cleaves the DNA backbone at abasic sites, aided by additional AP endonucleases, APE1L and APE2. BER in plants occurs via short-patch or long-patch pathways. Unlike animals, plants lack POLβ and instead utilize POLλ, which shows DNA polymerase and 5′-dRP lyase activities and interacts with PCNA homologs. POLδ also contributes to long-patch BER. FEN1 homologs provide flap endonuclease activity that is crucial for processing displaced DNA flaps, while AtLIG1 is the only ligase that can seal DNA nicks during repair. See text for abbreviations.

In Arabidopsis, BER proceeds via either short-patch or long-patch pathways (Córdoba-Cañero *et al*., 2009). The excision of 8-oxoG by OGG1 is preferentially followed by short-patch BER (Dianov *et al.*, 1998; Fortini *et al.*, 1999). Unlike animals, plants lack POLβ but possess a functional POLλ, which retains both DNA polymerase and 5′-dRP lyase activities (García-Díaz *et al*., 2000). In rice, POLλ exhibits polymerase, terminal transferase, and dRP lyase activities, interacts with PCNA, and can functionally substitute for POLβ (Uchiyama *et al.*, 2004). In Arabidopsis, POLλ, which is induced by UV, accurately pairs 8-oxoG with C. Of the two PCNA isoforms present, only PCNA2 interacts with POLλ (Amoroso *et al.*, 2011; Roy *et al.*, 2011; Morales-Ruiz *et al.*, 2024). Moreover, plants have FEN1 homologs that show both double-flap endonuclease and gap-dependent endonuclease activities. In Arabidopsis, *fen1* mutants demonstrate increased sensitivity to DNA damage induced by UVC radiation (Kimura *et al.*, 1997, 2000; Y. Zhang *et al*., 2016; J. Zhang *et al*., 2016). In rice, FEN1 interacts with PCNA, and this enhances the flap endonuclease activity of FEN1 (Kimura *et al.*, 2001). DNA ligase III is not present in plants; in Arabidopsis DNA ligase I (LIG1) is the only ligase involved in both short- and long-patch BER activities (Córdoba-Cañero *et al.*, 2011). XRCC1 homologs have been identified in Arabidopsis and rice. The interaction of XRCC1 with PCNA in rice suggests a conserved role in coordinating the BER machinery (Uchiyama *et al.*, 2008; Martínez-Macías *et al.*, 2013) (Fig. 6).

### Double-strand break repair

**Fig. 7.**
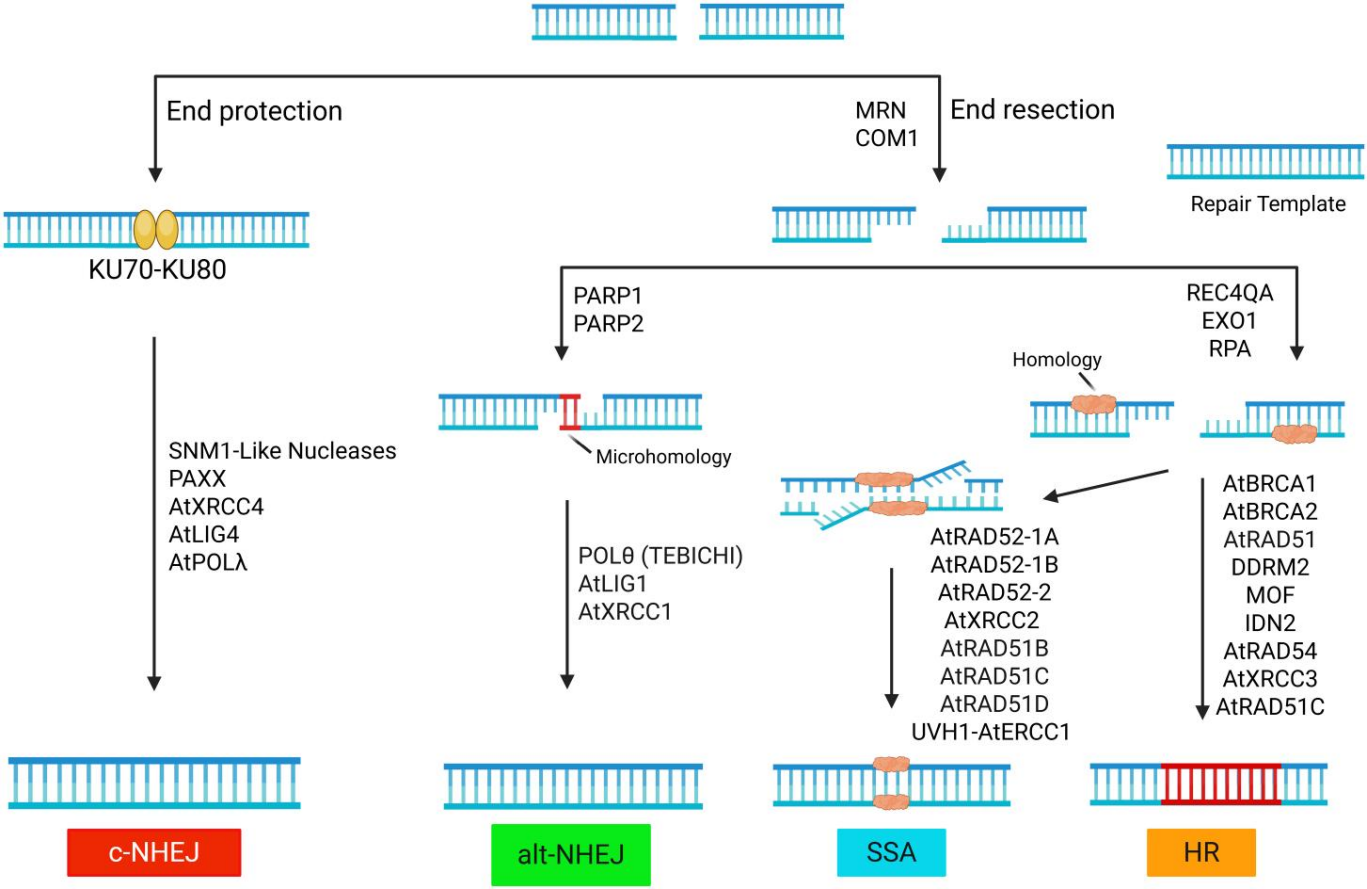
Double-strand break (DSB) repair pathways in plants. Plants employ four major pathways to repair DSBs: classical non-homologous end-joining (c-NHEJ), and three homology-directed repair (HDR) pathways, namely alternative non-homologous end-joining (alt-NHEJ), single-strand annealing (SSA), and homologous recombination (HR). In c-NHEJ, although plants lack DNA-PKcs and XLF, they possess a functional homolog of PAXX. SNM1-like nucleases and AtLIG4 may facilitate end processing, while AtPOLλ fills short DNA gaps. The three HDR pathways all begin with 5′–3′ resection of DSB ends, initiated by the MRN complex (MRE11–RAD50–NBS1) and COM1. In alt-NHEJ, PARP1 and PARP2 recognize DSBs and recruit repair proteins. POLθ (TEBICHI) promotes the annealing of microhomologous sequences and synthesizes DNA across the gap, leading to error-prone repair. AtLIG1 ligates the DNA ends, while AtXRCC1 serves as a scaffold protein that interacts with AtLIG1 to coordinate the repair process. SSA and HR both require extensive DNA end resection beyond the initial processing, and this is mediated by EXO1 and RECQ4A. In HR, the resulting ssDNA is initially bound by the RPA complex to protect it and prevent formation of secondary structures. AtBRCA1 and AtBRCA2 then facilitate the loading of AtRAD51 onto the ssDNA, a critical step that is supported by DDRM2, MOF, and IDN2. AtRAD51 catalyses strand invasion and D-loop formation, enabling homology search and strand exchange. AtRAD54 promotes the resolution of recombination intermediates. In addition, AtXRCC3 and AtRAD51C form a complex that stabilizes the AtRAD51 filament. In SSA, complementary ssDNA regions are annealed by RAD52-like proteins (AtRAD52-1A/B), while RAD51 paralogs (AtXRCC2, AtRAD51B, and AtRAD51D) also contribute to the process. Non-homologous flaps are removed by the UVH1–AtERCC1 endonuclease complex to complete repair. See text for abbreviations.

DSB repair can be either error-prone or error-free, depending on the pathway involved; however, in plants, a large proportion of DSBs are accurately repaired without introducing mutations (Ben-Tov *et al.*, 2024). Mechanistic insights into DSB repair pathways have mainly come from studies in humans and yeast. DSBs can be repaired by different pathways, including classical non-homologous end-joining (c-NHEJ), alternative non-homologous end-joining (alt-NHEJ), single-strand annealing (SSA), and homologous recombination (HR), each varying in its mechanism and fidelity (Fig. 7). The choice among these pathways depends on the extent of DNA end resection at DSB sites. When resection is minimal, c-NHEJ is preferred, as the KU70–KU80 heterodimer binds to and protects the ends of the DSB. This complex, consisting of DNA-Dependent Protein Kinase Catalytic Subunits (DNA-PKcs) and the endonuclease Artemis, performs limited end processing (typically <20 nucleotides), which is adequate for c-NHEJ. In contrast, HR, alt-NHEJ, and SSA, as types of homology-directed repair, require extensive 5′ to 3′ exonucleolytic resection at DSB sites. This process is initiated by the MRN complex, consisting of Meiotic Recombination 11 (MRE11), Radiation Sensitive 50 (RAD50), and Nijmegen Breakage Syndrome 1 (NBS1), in collaboration with C-terminal Binding Protein Interacting Protein (CtIP), which acts as a co-factor to enhance the endonuclease activity of MRE11 within the MRN complex. This process produces single-stranded DNA (ssDNA), which is necessary for these resection-dependent repair pathways. HR, which relies on a homologous template, is generally precise and preserves genomic integrity. In contrast, c-NHEJ, alt-NHEJ, and SSA are inherently error-prone and can lead to genomic rearrangements, such as insertions, deletions, or translocations (Chapman *et al.*, 2012; Ceccaldi *et al.*, 2016; Rogers *et al.*, 2025).

The choice of DNA repair pathways is regulated throughout the cell cycle. c-NHEJ remains active throughout the cell cycle but dominates in the G1 phase. On the other hand, HR becomes the preferred mechanism in the S phase, peaking in mid-S, which coincides with the peak of DNA replication. In the G2 phase c-NHEJ serves as the main pathway for repairing DSBs, similar to G1, whilst HR is necessary to repair only ∼15% of DSBs (Mao *et al.*, 2008; Beucher *et al.*, 2009). Comparison of plant and mammalian systems has shown that while mammalian cells perform accurate DSB repair in ∼50–55% of instances, tobacco cells achieve only 15–30% precision rates. DSB repair in plants also yields deletions and insertions longer than those found in mammalian cells (Gorbunova and Levy, 1997; Pelczar *et al.*, 2003).

#### Classical non-homologous end-joining

The predominant pathway in plants is c-NHEJ, an error-prone process characterized by inserting and deleting DNA segments at repair junctions, which may originate from nearby genomic regions or ectopic sites. c-NHEJ begins with the KU70–KU80 heterodimer recognizing and binding to the DSB ends. DNA-PKcs and the endonuclease Artemis assist in limited end processing, while POLλ and POLµ contribute to gap filling. The final ligation is performed by a complex consisting of XRCC4, XRCC4-like factor (XLF), PAXX (paralogue of XRCC4 and XLF), and DNA ligase IV (Chang *et al.*, 2017).

In Arabidopsis, KU70 and KU80 form a stable heterodimer with DNA end-binding activity at DSBs. The absence of KU70, KU80, and LIG4 results in hypersensitivity to DSB-inducing agents and reduces the efficiency of c-NHEJ (Bundock *et al.*, 2002; Riha *et al.*, 2002; Tamura *et al.*, 2002; West *et al.*, 2002; Gallego *et al.*, 2003). LIG4, which ligates the two ends of DSBs, interacts with the XRCC4 protein (West *et al.*, 2000; Friesner and Britt, 2003; van Attikum *et al.*, 2003). Plants lack homologs of core c-NHEJ factors such as DNA-PKcs and XLF; however, a plant PAXX homolog has been identified and shown to interact with Arabidopsis KU70, KU80, and XRCC4 (Khan and Ochi, 2023). Artemis belongs to the SNM1 nuclease family and contains conserved Metallo-β-Lactamase (MBL) and β-CASP domains, which are characteristic of this nuclease family. The genome of Arabidopsis encodes three SNM1-like proteins that possess conserved structural domains, suggesting they may perform roles similar to those of Artemis in plant c-NHEJ (Moshous *et al.*, 2001). Moreover, Arabidopsis LIG4 also contains a predicted β-CASP motif, potentially suggesting endonucleolytic activity reminiscent of Artemis (Bonatto *et al*., 2005). The only X family polymerase experimentally shown to repair DSBs in the genome via c-NHEJ is Arabidopsis POLλ, which interacts with XRCC4 and LIG4 through its BRCA1 C-terminal (BRCT) domain. Transgenic *polλ* mutant lines expressing a BRCT-deficient POLλ protein show increased sensitivity to DSB-inducing agents (Roy *et al.*, 2013; Furukawa *et al.*, 2015).

#### Alternative non-homologous end-joining

The c-NHEJ and alt-NHEJ pathways can effectively compensate for each other in DSB repair (Merker *et al.*, 2024); however, the alt-NHEJ pathway is inherently more mutagenic. Both c-NHEJ and HR actively inhibit alt-NHEJ (Ahrabi *et al.*, 2016; Shen *et al.*, 2017). alt-NHEJ activity is associated with the XRCC1–DNA ligase III complex and the Poly(ADP-ribose) Polymerase 1 (PARP1) enzyme, which competes with the KU heterodimer for binding to DNA ends (Wang *et al.*, 2006; Charbonnel *et al.*, 2010). alt-NHEJ utilizes short microhomologies ranging from 2–20 bp near the DSB for repair via POLQ, a non-replicative polymerase involved in DSB repair. In this process, POLQ is recruited to the resected DSB ends, facilitating the annealing of the short homologous regions and the subsequent DNA synthesis to complete the remaining gaps (Kent *et al.*, 2015; Zahn *et al.*, 2015; Black *et al.*, 2016; Wood and Doublié, 2016). POLθ facilitates insertion mutations during alt-NHEJ by acting as a terminal transferase, which adds nucleotides directly to the 3′-ends of DNA at DSBs (Kent *et al.*, 2016). Through its helicase domain, POLθ removes RPA from resected DSBs, enabling their annealing and eventual joining through alt-NHEJ. Consistent with the opposing function of RPA during alt-NHEJ, inhibiting RPA boosts end-joining while reducing recombination (Mateos-Gomez *et al.*, 2017).

Homologs of PARP1 and PARP2 are present in plants, where their transcription is induced by oxidative stress. These proteins localize to the nucleus, and their poly(ADP-ribosyl)ation activity is stimulated by DNA strand breaks. (Lepiniec *et al.*, 1995; Babiychuk *et al.*, 1998; Doucet-Chabeaud *et al.*, 2001; Jia *et al.*, 2013). POLθ (TEBICHI in Arabidopsis) plays a critical role in plant genome stability and development. *teb* null mutants display severe developmental defects, such as reduced growth, deformed leaves, disorganized root meristems, activation of constitutive DNA damage responses, and hypersensitivity to UV. Mutations in several alleles, including *teb1*, *teb2*, and *teb5*, exhibit similar phenotypes. In contrast, *polQ* mutants in the moss *Physcomitrium patens* show no developmental defects, although they are DSB repair-deficient. *PolQ* mutants in rice have been shown to have normal growth under standard conditions, regeneration from callus was severely impaired, and some developmental abnormalities were observed (Inagaki *et al.*, 2006, 2009; van Kregten *et al.*, 2016; Mara *et al.*, 2019; Nisa *et al.*, 2021; Nishizawa-Yokoi *et al.*, 2021; Kralemann *et al.*, 2022).

Plants appear to compensate for the lack of ligase III by utilizing DNA ligase I and possibly other ligase enzymes. The absence of LIG1 in Arabidopsis causes lethality during embryonic development, highlighting its critical role in plant growth. A decrease in *LIG1* expression leads to slower repair of both SSBs and DSBs, suggesting that the protein is involved in the repair processes for these two types of DNA damage. Arabidopsis has an ortholog of XRCC1, but it interacts primarily with DNA ligase I, unlike the XRCC1–ligase III complex in mammals (Waterworth *et al.*, 2009; Martínez-Macías *et al.*, 2013).

#### Homologous recombination

The MRN complex triggers the homology-directed repair pathways. When homologous DNA sequences such as sister chromatids or homologous chromosomes are available as repair templates, the MRN complex, along with CtIP, forms a nuclease complex that resects the 5′-strands at the DSB site, resulting in 3′-ssDNA overhangs. These 3'-overhangs are protected by RNA–DNA hybrids, which are mediated by RNA polymerase III (RNAPIII) and recruited to DSBs by the MRN complex. Further resection is carried out by the Exonuclease 1 (EXO1) and Bloom Syndrome Protein (BLM) helicase-DNA2 nuclease complex, whose activities are enhanced by the Breast Cancer Type 1 Susceptibility Protein (BRCA1)–BRCA1 Associated RING Domain Protein 1 (BARD1) E3 ubiquitin ligase complex. These overhangs are quickly coated by RPA, stabilizing the ssDNA and preventing secondary structure formation. BRCA2 then facilitates the replacement of RPA with the DNA recombinase RAD51, leading to the formation of RAD51–ssDNA nucleoprotein filaments. These filaments facilitate strand invasion into a homologous DNA duplex, forming a displacement loop (D-loop). RAD51 loading at the DSB site and stabilization of RAD51 microfilament are mediated by RAD54, a motor protein belonging to the SWI2/SNF2 helicase family that translocates along dsDNA. PALB2 binds directly to both BRCA1 and BRCA2, coordinating the DNA end resection step with the RAD51 filament formation. The D-loop acts as a primer for DNA synthesis, using the undamaged homologous sequence as a template, thus enabling high-fidelity DSB repair (Xia *et al.*, 2006; Sy *et al.*, 2009; Ma *et al.*, 2017; Zhao *et al.*, 2017; Wright *et al.*, 2018; Liu *et al.*, 2021; Whelan and Rothenberg, 2021; Salunkhe *et al.*, 2024).

Like other eukaryotes, plants rely on the MRN complex for DSB repair. In rice, MRE11 is widely expressed and its loss causes severe defects in growth, gametogenesis, and seed development; it forms a complex with RAD50 and NBS1 (Ji *et al.*, 2013; Shen *et al.*, 2020; Nair *et al.*, 2021). In Arabidopsis, MRE11 and RAD50 are required for DSB repair, and mutant plants are more sensitive to DNA damage and have various defective developmental phenotypes (Gallego *et al.*, 2001; Bundock and Hooykaas, 2002; Puizina *et al.*, 2004; Amiard *et al.*, 2010; Šamanić *et al.*, 2016). In *P. patens*, MRE11 binds to NBS1, and mutants lacking MRE11 and RAD50 exhibit growth defects and increased sensitivity to genotoxic stress, whereas *nbs1* mutants do not show these phenotypes (Reference). Similarly, NBS1-deficient Arabidopsis plants are viable and develop normally (Kamisugi *et al.*, 2012; Najdekrova and Siroky, 2012; Das *et al.*, 2025). MRE11 and RAD50 have been shown to interact in Arabidopsis extracts (Daoudal-Cotterell *et al.*, 2002), and wheat MRE11 also interacts with RAD50, indicating similar functions in monocot plants (Pérez *et al.*, 2011). Furthermore, COM1, the plant homolog of human CtIP, influences the 5′–3′ nucleolytic resection activity of the MRN complex (Andres and Williams, 2017). In Arabidopsis, MRN activity is regulated by COM1, and its absence leads to sensitivity to DSB-inducing agents, results in infertile plants, and causes defects in the formation of RAD51 foci, despite the presence of unrepaired DSBs (Uanschou *et al*., 2007). Similarly, maize plants lacking Com1 exhibit deficiencies in RAD51 loading at DSBs (Wang *et al*., 2018). In rice, COM1 provides resistance to DNA-damaging agents. The Meiotic F-Box (MOF) also plays an important role in the recruitment of COM1 and RAD51C to DSB sites (Ji *et al*., 2012; He *et al*., 2016; Xu *et al*., 2020).

In Arabidopsis, the RecQ helicase RECQ4A is the functional homolog of human BLM. The lack of RECQ4A leads to increased sensitivity to DNA-damaging agents, underscoring the crucial role it plays in maintaining genome stability (Bagherieh-Najjar *et al.*, 2003; Hartung *et al.*, 2007; Schröpfer *et al.*, 2014). RECQL4 plays a similar role in rice, as *recql4* mutants exhibit high sensitivity to genotoxic stress. Overexpressing *RECQL4* and *EXO1* (homolog of the human gene of the same name) enhances the resection of DSB ends, thereby improving the efficiency of HR-mediated repair (Kwon *et al.*, 2012, 2013). The RPA complex is a heterotrimer composed of RPA1, RPA2, and RPA3 subunits. The Arabidopsis genome encodes five RPA1 homologs (A–E), two RPA2 homologs (A and B), and two RPA3 homologs (A and B). Of these, RPA2B forms a functional complex with either RPA1C or RPA1E and, together with RPA3A or RPA3B, plays a crucial role in HR-mediated DSB repair, as indicated by the observation that *rpa2b*, *rpa1c*, *rpa1e*, and *rpa3a* mutants show reduced HR repair efficiency (Liu *et al.*, 2017; Aklilu *et al.*, 2020). Moreover, EXO1 in rice interacts with RPA70b, RPA32-1, and RPA14 (Ishibashi *et al.*, 2006; Furukawa *et al.*, 2008). Arabidopsis contains an ortholog of BRCA1 as well as two functional BRCA2 homologs. Double-mutants of these BRCA2 homologs are hypersensitive to agents that induce DSBs and show a significant reduction in HR after DSB induction. Both the BRCA2 proteins interact with RAD51, and the absence of either one impairs RAD51 localization (Lafarge and Montané, 2003; Dray *et al.*, 2006; Seeliger *et al.*, 2012). Arabidopsis RAD51 is highly conserved and functionally analogous to human RAD51. Its expression is up-regulated in response to DSB-inducing agents and is induced during the S-phase of the cell cycle. The absence of RAD51 in Arabidopsis disrupts HR, resulting in higher sensitivity to genotoxic stress. Arabidopsis also contains several RAD51 paralogs. Among these, XRCC3 and RAD51C form a complex that facilitates RAD51 filament stabilization and D-loop formation during strand invasion. Mutants lacking either gene are hypersensitive to DSB-inducing agents and show reduced HR efficiency. XRCC3 similarly supports DSB repair in rice, suggesting a conserved role across plant species (Doutriaux *et al.*, 1998; Osakabe *et al.*, 2002; Bleuyard and White, 2004; Li *et al.*, 2004; Abe *et al.*, 2005; Zhang *et al.*, 2015). Efficient repair of DSBs in Arabidopsis depends on the proper recruitment of RAD51 to the DSB sites, coordinated by multiple accessory factors. One such factor is DNA Damage Response Mutant 2 (DDRM2) protein, which directly interacts with RAD51 and is essential for its localization at DSB sites (Yu *et al.*, 2023). MOF also facilitates the recruitment of COM1 and RAD51C to DSBs (He *et al.*, 2016). In addition, the RNA-binding protein IDN2 interacts with the RPA complex to promote RAD51 loading by facilitating the displacement of RPA from ssDNA (Liu *et al.*, 2017). Lack of RAD54 results in increased sensitivity to DSB-inducing agents, and it forms subnuclear foci following the induction of DSBs (Osakabe *et al.*, 2006; Hirakawa *et al.*, 2017; Hirakawa and Matsunaga, 2019).

#### Single-strand annealing

This is a RAD51-independent, error-prone form of homology-directed repair that does not require an external donor template. Following a DSB between repeated sequences, extensive end resection by the MRN complex, CtIP, EXO1, and the BLM–DNA2 complex results in homologous repeat regions. These complementary repeats anneal directly without strand invasion, mediated by RAD52 proteins. The resulting non-homologous 3′-ssDNA flaps are cleaved by the XPF–ERCC1 endonuclease complex, followed by gap filling and ligation. This process leads to the deletion of the intervening DNA sequence, rendering SSA a mutagenic yet efficient strategy for repairing DSBs in repeat-rich genomic regions. (Ivanov *et al.*, 1996; Al-Minawi *et al.*, 2008; Scully *et al.*, 2019; Gallagher and Haber, 2021). The SSA pathway is conserved in plants, where RAD52-like proteins mediate annealing between homologous repeat sequences during DSB repair. In Arabidopsis, two RAD52 homologs exist, RAD52-1 and RAD52-2, and the expression of *AtRAD52-1A* partially complements DSB repair defects in the yeast *rad52* mutant. Loss of either RAD52-1 or RAD52-2 leads to increased sensitivity to DNA-damaging agents and a reduction in the efficiency of intrachromosomal HR (Samach *et al.*, 2011). In addition to RAD52, the Arabidopsis RAD51 paralogs XRCC2, RAD51B, and RAD51D are also involved in the RAD51-independent SSA pathway, with RAD51C serving as a scaffold for the recruitment of RAD51B to the DSB site (Serra *et al.*, 2013; Xu *et al.*, 2018). The *uvh1* and *ercc1* mutants in Arabidopsis are hypersensitive to ionizing radiation, implying the essential role of the UVH1–ERCC1 complex in cleaving non-homologous 3′- or 5′-ssDNA tails during SSA (Gallego *et al.*, 2000; Dubest *et al.*, 2002, 2004; Preuss and Britt, 2003; Guyon-Debast *et al.*, 2019). In rice, RAD52-2A binds both single- and double-stranded DNA, and mediates the annealing of complementary ssDNA to reconstitute dsDNA during SSA-mediated DSB repair (Nair *et al.*, 2016).

## Conclusions and future prospects

The mechanisms for UV tolerance in plants involve shielding with flavonoid molecules, repairing damage by photoreactivation and nucleotide excision repair, and maintaining DNA replication with the help of TLS polymerases, which can bypass UV photoproducts. UV-generated ROS and replication stress also cause oxidative damage and DSBs. To eliminate these DNA lesions, plants use BER and various DSB repair pathways, including c-NHEJ, alt-NHEJ, HR, and SSA. These protective mechanisms and repair pathways work together to maintain genome stability and continuity in the presence of UV stress. There is increasing interest in understanding how plants cope with UV stress and in the development of tolerant cultivars, prompted by continuing concerns over increasing levels of harmful UV radiation reaching the Earth's surface due to ozone-layer depletion and global warming, the idea of farming in space(especially on Mars) where crops are exposed to higher UV levels than on Earth, the need to maintain genome stability in high-yielding crops, and the desire to understand the evolution of UV tolerance mechanisms during plant terrestrialization.

Despite valuable advances in understanding plant UV-tolerance mechanisms, significant gaps remain in this field. A considerable challenge in terms of plant DNA repair is the limited number of mechanistic studies aimed at elucidating the molecular basis of UV tolerance, particularly the pathways involved. Compared with organisms such as yeast or mammals, several core DNA repair factors are absent in plants, while others appear to be duplicated or diversified. This implies that they might employ unique, plant-specific DNA repair mechanisms; however, our understanding of these pathways remains largely incomplete due to the scarcity of detailed molecular and biochemical analyses, particularly those that define protein–protein and protein–DNA interactions. Most of our current knowledge is derived from genetic studies, mutant phenotyping, gene-expression profiling, and epistasis analysis, which—while informative–are insufficient to resolve the precise molecular architecture and dynamics of repair complexes. Advancing this field will require targeted efforts to dissect the molecular interactions and structural features of plant DNA repair factors, enabling a clearer understanding of how genome stability is maintained under UV stress.

One of the unresolved questions regarding the repair of UV-induced DNA damage in plants involves the rationale behind the fact that they exhibit both photoreactivation and nucleotide excision repair. What are the specific functions of these two mechanisms, and how do they support one another in maintaining genome integrity? Another compelling question centers on how plants can execute NER in the absence of a homolog of XPA, a protein crucial for efficient nucleotide excision repair in other eukaryotic organisms. The exact molecular mechanisms through which plants compensate for the absence of XPA remain unidentified. Moreover, our understanding of DNA repair dynamics at the single-cell level is still limited. The introduction of XR-seq technology has made the generation of genome-wide repair profiles feasible; however, it is crucial to utilize it at a single-cell level in order to comprehend how each plant cell reacts to and repairs UV-induced DNA damage. Another prominent gap in our knowledge relates to the mechanisms of DNA damage repair within heterochromatic regions. The chromatin landscape serves as an obstacle to repair processes, raising questions regarding the dynamic nature of chromatin structure and the specific factors governing its compaction and decompaction during nucleotide excision repair. Addressing these points is essential for a thorough understanding of the protective strategies employed by plants against UV-induced genotoxic stress. Research focusing on these areas will not only help us bridge the gaps in our knowledge of the plant DNA repair domain but also help with the development of crop varieties with enhanced resilience to UV-induced DNA damage.

## Data Availability

No new data were generated or analyzed in this study. All data discussed are from previously published sources, which are cited in the manuscript.
